# High-Efficiency Multi-Sensor System for Chair Usage Detection

**DOI:** 10.3390/s21227580

**Published:** 2021-11-15

**Authors:** Alessandro Baserga, Federico Grandi, Andrea Masciadri, Sara Comai, Fabio Salice

**Affiliations:** 1 Department of Physics, Politecnico di Milano, 20133 Milan, Italy; alessandro.baserga@mail.polimi.it (A.B.); federico.grandi@mail.polimi.it (F.G.); 2 Department of Electronics, Information and Bioengineering, Politecnico di Milano, 20133 Milano, Italy; andrea.masciadri@polimi.it (A.M.); fabio.salice@polimi.it (F.S.)

**Keywords:** fall detection, chair usage, ambient assisted living, capacitive coupling sensor, accelerometer sensor, Activities of Daily Living

## Abstract

Recognizing Activities of Daily Living (ADL) or detecting falls in domestic environments require monitoring the movements and positions of a person. Several approaches use wearable devices or cameras, especially for fall detection, but they are considered intrusive by many users. To support such activities in an unobtrusive way, ambient-based solutions are available (e.g., based on PIRs, contact sensors, etc.). In this paper, we focus on the problem of sitting detection exploiting only unobtrusive sensors. In fact, sitting detection can be useful to understand the position of the user in many activities of the daily routines. While identifying sitting/lying on a sofa or bed is reasonably simple with pressure sensors, detecting whether a person is sitting on a chair is an open problem due to the natural chair position volatility. This paper proposes a reliable, not invasive and energetically sustainable system that can be used on chairs already present in the home. In particular, the proposed solution fuses the data of an accelerometer and a capacitive coupling sensor to understand if a person is sitting or not, discriminating the case of objects left on the chair. The results obtained in a real environment setting show an accuracy of 98.6% and a precision of 95%.

## 1. Introduction

Data collected by the United Nations show a clear trend regarding ageing society: in 2050, the global average age will reach the value of 38 years old, while it was barely 26 in 1990 [1]. According to research conducted by the University of Washington, 2.4 billion people will be 65 years old or older, and only 1.7 billion will be 20 years old or less [2]. This ageing society is a global trend hitting the developed world particularly hard, in which the population of age over 85 will quadruple by 2050 with respect to today’s figures [3]. Healthcare facilities all over the world are faced with the task of taking care of a rising percentage of the population and a drastic change in paradigm is long due to prevent the collapse of the system. Technology favours this change, promoting a preventive-care paradigm of welfare through monitoring tools that allow assessing the individual’s well-being and anticipating undesirable situations with corrective actions [4,5], possibly exploiting artificial intelligence techniques [6]. This concept requires the ability to monitor and assist people in their everyday lives, especially at their own homes as a measure to reduce healthcare costs in hospitalizations [7,8]. The goal is to create a “proactive healthcare system” that tries to keep the patients healthy, encouraging the maintenance of physical and cognitive abilities through the execution of specific daily activities, thus avoiding the need for a caregiver in everyday tasks.

Several home care monitoring systems have been proposed where IoT sensors are used to understand the Activities of Daily Living of the persons, their positions, their routines; they possibly provide alerts to families or caregivers when anomalies occur (e.g., [9,10,11,12]). Several solutions monitor daily activities of seniors to prevent critical situations such as falls. In fact, falls represent one of the major health hazards among the population of age over 60 living alone and are considered the eighth leading cause of death in the U.S. [13]. If a person falls unconsciously without getting emergency treatments, irreversible consequences such as fracture, stroke, disability and even death may occur. Moreover, being unable to get up and thus lying on the floor for a long time (this event is called a “long lie”) can lead to serious injuries, admissions to hospital, and subsequent moves into long term care [14]. Considering the importance of the problem, different techniques have been proposed for fall detection in the literature, from wearable devices, to the use of cameras or IoT and sensor fusion [15,16]. However, cameras and also wearable devices are considered intrusive by many users. To support human activity recognition in an unobtrusiveway, ambient-based solutions can be adopted using sensing systems far away from the human body (device-free) [17].

In this paper, we focus on *chair usage detection*, exploiting only unobtrusive sensors. Sitting detection is important to solve different problems such as monitoring activities where the person is sitting at the table or supporting fall detection algorithms. Considering the latter aspect, our vision is based on the following assumption: it is highly unlikely that a person will intentionally spend much time *stationary* in the same position at home unless he/she is *sitting*, *lying down* or has *fallen*. If we can track a person inside their domestic environment, stationary positions do not pose any problem if the person is sitting on a chair or sofa or is lying in their bed; instead, a stationary and prolonged presence in a room where the person is not sitting/lying down increases the probability that the person has fallen as time passes in which no further movements are recorded.

While identifying a seat in a sofa or bed in an unobtrusive way is reasonably simple (e.g., with bed pressure sensors) and, above all, has no significant power supply problems due to its natural static position, detecting whether a person is sitting on a chair is still an open problem due to the natural chair position volatility.

At this aim, we propose a sitting detection system that has no impact on the chair already present in the home, that is not invasive, not energetically wasteful, and reliable. The system is based on an accelerometer and a capacitive coupling sensor: this allows us to understand if a person is sitting or not, but also to discriminate the case of other objects left on the chair. The system has been tested in a real environment setting.

The rest of the paper is organized as follows: after reviewing related work in Section 2, Section 3 describes the requirements of the system, the proposed architecture and the implemented algorithm, while Section 4 reports the data collected in the testing phase with different configurations of the system, and discusses the results. Finally, Section 5 draws the conclusions and reports future developments of the proposed solution.

## 2. Related Work

The ability to detect the sitting behaviour of a human being has been widely studied in relation to posture detection to improve people’s conditions in workplaces. Three main types of posture measurement techniques are typically used: many proposals exploit image processing technology, some exploit wearable devices; others are based on ambient-based methods and rely on pressure sensors, passive infrared motion sensors, etc.

**Camera-based solutions**. Posture detection by means of cameras have been used for several applications. Lan et al. [18] proposed a surveillance system for the human sitting posture with the goal of preventing myopia and backbone/neck diseases caused by the use of computers; first, the profile of sitting posture is extracted, then, by comparing the real-time profile features with the standard profile features, the surveillance system will remind the user to correct their sitting posture. Song-Lin et al. [19] proposed a system that exploits PCA and facial motion recognition to detect eight typical sitting postures.

Approaches like these rely on the use of a camera placed in front of the user, which raises some particular privacy concerns, and requires some costly hardware. Moreover, they are intended to be used mainly in environments that tend to be stationary.

**Wearable devices**. The posture of the person can be identified also by means of wearable devices that use different kinds of sensors. Mekruksavanich et al. [20] recognize periods of sitting at work with the aim of identifying the Office Workers Syndrome where the worker is sitting for a long time at the desk. They exploit smartwatch sensors, in particular accelerometers and gyroscopes, and apply ensemble learning techniques. The recognition capability achieved an accuracy level of 93.57 percent. Fuller et al. [21] examined Apple Watch and Fitbit to check if commercial wearable devices can accurately predict lying, sitting and varying intensities of walking and running. Considering the sitting activity, classification accuracy for Apple Watch was 72.6% and for Fitbit 86.2%. Nyan et al. [22] created a garment as a wearable platform equipped with a MEMS technology-based triaxial accelerometer that was attached at the shoulder position of the garment. The system monitored different user’s activities such as walking, sitting down, standing up and also falls. The focus is more on transitions from sitting postures to lying/standing postures. For all the activities they obtained an average sensitivity of 94.98%. Finally, Sazonov et al. [23] use a shoe sensor to classify the different posture activities. Using a combination of acceleration/pressure sensors, they classify sitting/standing postures and walking, running, stair ascent/descent and cycling activities using Support vector machines (SVMs) with a 95.2% average accuracy. Wearable devices can be used with good results to detect the different postures. These devices are cheap, and easy to set up and operate. However, this approach is considered intrusive: most of the users dislike wearing a device or simply forget to wear them.

**Ambient-based methods**. A third group of solutions consider unobtrusive sensors. Huang et al. [24] proposed an architecture for information capture and analysis of sitting posture using *force sensors*. Those were placed in fixed positions on seat cushions to allow the system to determine the different sitting postures. This system could detect incorrect sitting postures for children, patients or elderly people. Zhu et al. [25] designed a sensing chair equipped with a commercially available *pressure* distribution sensor, that is optimal for high resolution and the flexibility of the sensor sheets so they can conform to the shape of a chair. The two sensor sheets were surface mounted on the seat pan and the backrest of the chair. Both these methods are very powerful in detecting human postures. However, some of these sensors modify the configuration and aesthetic of the chair which could be a negative factor in an elder user life scenario. Moreover, there is not a clear way to discriminate what kind of body is pressing onto the sensor, which can lead to some false identification of extraneous bodies. Ma, C. et al. [26] implemented the idea of a “smart cushion” to monitor the activity level of wheelchair users. The system is composed of six *pressure* sensors, one *inertial measurement unit*, and a Bluetooth communication unit connected to a Micro Controller Unit (MCU). The smart cushion can vibrate to alert the user to engage in some physical activity in case of an excessive sedentary routine. The article reports an accuracy of 89% for activity recognition and 98% for activity level recognition. The system can perform complex data analysis, but requires more advanced, and in turn more expensive, hardware. Our solution is simpler, does not aim at detecting fine activities on the chair, but is thought to be integrated into a smart home and to be deployed on several chairs. Rosato [27] proposed a different system: the idea is to exploit Bluetooth Low Energy (BLE) beam technology for proximity detection. The system is made of one or multiple couples of an antenna and a receiver under the chair and measures the amount of distortion in the signal when a human being is present between the beacon and the receiver. Their results reached a precision up to 94% with multiple beacons, but, on the other hand, every situation required a single analysis to reach the optimal configuration. This last system highly depends on environmental conditions and if the chair is moved, the detection gets significantly worse. Moreover, the use of multiple beams can result in great power consumption, hindering the lifetime of the system. In conclusion, most of the works related to sitting detection propose highly specific systems with a defined purpose, such as posture detection, which is way more advanced than the binary sit–not sit information supported by the system proposed in this paper, but do not satisfy other important requirements such as the possibility of moving the chairs or the unobtrusiveness or the possibility to deploy it on several chairs thus with a low cost.

## 3. Materials and Methods

In this section, we describe the requirements of the proposed system, the analysis of the possible sensors to be used for the identified requirements, its architecture and the algorithm used for detection. Given that the idea is that the chair should be freely moved inside the domestic environment without requiring cables or changing furniture, power consumption issues have also been considered.

### 3.1. Requirements Specifications

The functional and non-functional requirements of the system were obtained from three brainstorming sessions about the limits of the alternatives available in the literature and the innovative paradigm chosen for the detection of falls.

- **Functionality:**
  The system should have the ability to detect a sitting person with a very low rate of false-positive cases (e.g., a bag that is placed on the seat should generate no alert).
- **Modularity:**
  Due to the presence of multiple chairs in a house, the detecting device should be modular and a single storing/transmitting unit per home should be used.
- **Connectivity:**
  Chairs are highly volatile objects in the house; the presence of cables to transmit data would render chairs dysfunctional, unpleasant to look at and may even enhance the risk of fall.
- **Power management:**
  As in the previous point, the use of power cables is not recommended; the use of batteries is required. However, for the system to be effective in fall detection, it must always be active and maintenance operations such as battery charging should be minimized. A battery life of more than one year is considered acceptable.
- **Non intrusiveness:**
  The system should not interfere with the daily habits of the person. In particular, it should not require a substantial modification to the commodities.
- **Non-disruptiveness:**
  The system should not require a radical change in the routine of the user (for instance, a wearable device).

### 3.2. Sensor Analysis

Several physical phenomena can be exploited in order to detect whether a person is sitting on a chair or not. To identify the best choice in terms of sensors, we analysed different alternative solutions that could satisfy the system requirements. In particular, we analysed: visible light detectors, infrared light sensors, sound detectors, mechanical detectors, accelerometers and capacitive sensors. In the following, we discuss their possible placements and their main advantages and disadvantages.

- Visible light sensing may be installed under the seat or on the backrest in order to detect the shadow of a person that is sitting down. However, this solution could be affected by the overall light condition of the room or the presence of non-human objects blocking the light impinging on the sensor. Moreover, the energy consumption of this type of device strongly relies on the amount of light detected.
- Infrared light sensing has similar issues to the light-sensing described above, especially referring to passive infrared detectors. Another choice could be the usage of a proximity detector in combination with an IR LED, these sensors offer great accuracy in detecting whether an object is approaching the chair but are not able to distinguish human beings from objects. Moreover, the IR LED increases significantly the energy consumption of these devices and they may infringe the sense of privacy of the user.
- Sound detectors could be both microphones or ultrasonic detectors. In the first case, one could decide to detect humans sitting by the sound generated. As in the visible light sensors, this type of detection is really affected by environmental noise and requires a specific circuit in order to obtain the correct amplification of the signal. Ultrasonic detectors behave in the same way as IR proximity sensors so they inherit the same issues regarding high energy consumption and the impossibility to unequivocally detect whether the measurement involves a human being or not.
- Weight sensors installed under the legs of the chair, could very precisely detect the sitting of a person and could possibly distinguish input given by bags that are much less heavy than a human being with an optimal calibration. The main drawback is the modification to the chair could be disliked by the user, and the high degradation under its legs caused by the usage will destroy the sensor in a very short amount of time.
- Accelerometers could be potentially installed nearly everywhere on the chair and will detect the sitting behaviour of the user measuring the natural vibration induced by the presence of a person on a chair with an appropriate resolution. Object detection can be avoided noting that they do not produce any vibration while resting on a chair. Energy consumption can be very low and it is generally possible to enable a trigger in order to run the system mainly in sleep mode.
- Capacitive sensing exploits the change in charge density induced by the presence of a person near the sensor, which could be placed under the seat or in the backrest. False-positive cases could arise with objects with high quantities of water, caused to their similarity with a human body in terms of dielectric properties. Detection can be achieved by microcontrollers or specific low energy chips capable of generating interrupt signals.

Sensors have been evaluated with respect to different characteristics deemed important for our system’s specifications: power consumption, intrusiveness, reliability, and price. For each aspect, we assigned a value among low, medium, high, according to our our previous experience with the analysed sensors. The results are summarized in Table 1. Light based sensors and ultrasound detectors are characterised by a higher energy consumption, leading to a very low autonomy. In the case of sound sensors, some ambient disturbances could greatly hinder the detection. Load cells could be a great candidate, but they suffer greatly in friction durability and would quickly wear down. Given the main requirements of the system, we selected capacitive and accelerometer sensors as key sensors of the proposed system.

Capacitive sensors seem to be the primary choice for a sitting detection system due to their sharp response to objects located on the chair. Indeed, different objects produce different capacitive data so the distinction between a human body and an inanimate object is, in principle, possible.

Nevertheless, an accelerometer could still be used to detect the starting point of usage of the chair, leaving then to the capacitive sensor the task of correctly understanding the sitting activity from that moment. A possible implementation could combine the information of the two sensors, using the accelerometer to generate an interrupt that activates the capacitive sensing providing a stronger power-saving feature. Considering that the reduction of the energy consumed by the devices is a requirement of the system, we compared this parameter in *operation state* and *energy saving state* for different capacitive and accelerometer sensors (Table 2).

### 3.3. General Architecture and Power Save Features

**Figure 1:**
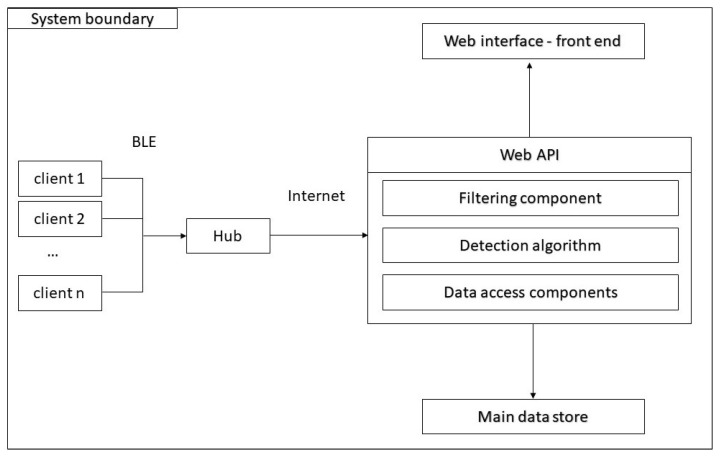
Final architecture of the system.

This section presents the general architecture of the system (see Figure 1), and discusses different power-saving techniques that may be useful to increase the battery life.

A client/server architecture has been implemented. The client is the object that is attached to the chairs and collects the data to be sent to a server for further analysis. It consists of a microcontroller, a sensing system and a communication device.

An ATTINY85-20PU microcontroller has been chosen due to its limited power consumption (300 µA in 1.8 V normal mode, 0.1 µA in 1.8 V power-down mode), quite good memory dimension (8 Kb of programmable memory, 0.5 Kb of EEPROM) and low price.

As a sensing plate, we chose a copper foil for its great conductive properties and cost. Moreover, the conductive glue allows great customisation in the possible geometries of the sensing apparatus with minimal energy dispersion and a reasonable benchmark for future implementation in a printed circuit board.

### 3.4. Power Consumption Considerations

Since battery life is one of the fundamental requirements of the system, the design has been greatly influenced by considerations regarding the client’s power savings. For example, the system has been designed to have all unnecessary components of the MCU not powered (f.i the ADC) and is programmed to remain in deep sleep mode until an interrupt is generated by an accelerometer (in particular the MMA8451) with an appropriate sensibility to pick up vibrations generated by the user while sitting. The frequency of the capacitive measure is gradually diminished using a discrete-time linear system, starting from a period of 100 ms and increasing up to 5 min exponentially, resetting upon the signal of the accelerometer interrupt. This allows the system to waste as little energy as possible.

The communication interface is the main power-draining component of the system and the choice of protocol and hardware is heavily influenced by the specifics of every house, such as square footage, wall presence and the number of devices used. A Bluetooth Low Energy (BLE) interface as the HM-19 would allow us to have a good trade-off in terms of energy consumption, range, price and multiplex capabilities. Moreover, in order to reduce power consumption, a watchdog system has been introduced in the communication protocol: the clients send data packets of variable dimensions with variable rates that are linked to the changing of chair usage. A packet is sent either if the system has collected 10 samples or 5 min have passed or the accelerometer has detected a sudden change in position. The packet acts as a “heartbeat” of the client, allowing the server to diagnose broken or malfunctioning clients if two or more 5 min windows of time elapse without receiving the heartbeat of a specific client. In addition, a packet approach greatly reduces communication energy consumption in comparison to other techniques.

The clients are programmed to send via BLE their data to a local HUB that transfers the data to a remote server through the internet. This hub consists of a receiver, an internet interface (possibly WLAN) and could be powered by a wall plug given the non-invasive nature of this device.

The hub sends the data of a single client to the Web API, where it is processed by various filters to reduce the noise as much as possible. The output is then fed to the decision algorithm that converts the data contained in the signal into information on whether someone is seated or not. This is all displayed by the web interface.

### 3.5. Algorithm and Calibration

The detection capabilities of the system are handled in the main server by a specific algorithm described in this paragraph.

The data extracted from the capacitive sensor while the chair is empty could be represented by Gaussian noise with nonzero mean thanks to its stochastic nature. When a body is placed on the chair, the mean of the signal increases considerably. During a calibration phase, the system will acquire some measurements of an empty chair to estimate the mean and the standard deviation of the process. Given these considerations, the algorithm divides the data into windows of N elements, checks if the data is above the mean plus three times the standard deviation obtained during calibration and assigns the status of that particular window as occupied whenever a given threshold percentage $n_{\mathrm{th}}$ of the elements is above that value. During some preliminary tests we found that setting the values of the parameters N = 5 and $n_{\mathrm{th}}$ = 0.7 yields consistent and reliable results. As stated above, the method requires a calibration phase to establish with a good degree of confidence the characteristics of the process while the chair is not used. To achieve this, the device needs to undergo a calibration phase in which a data sample of at least 100 elements is collected. The estimated time to complete this test is estimated to be around 2 min. This phase should not be considered critical, because when properly installed, the system does not need any hard skill to be calibrated.

A possible way to increase the accuracy of the system is to add to the algorithm a check on the intensity of the signal while a person is seated, in order to reduce the false positives. In order to archive this, a second part of the calibration phase should be undertaken, in which the final user sits on the chair to allow the system to collect a meaningful amount of data. This option was not included in the final system in light of the fact that it would complicate the set-up phase in an excessive way.

## 4. Results and Discussion

Multiple studies on the sensor design and the system performance have been carried out. The first part of this section focuses on finding the best geometric configuration of the sensing plate in terms of both performance and energy consumption. The second part presents a performance test on a real-life use scenario and identifies the possible limitations of the system. Finally, the third part discusses the estimation of the energy consumption of the system during its activity and some other possible adjustments that could increase performance and battery life.

In our experiment, the sketch client was a NodeMCU V1.0 connected by a Wi-Fi link to a remote server able to collect data in a CSV file. The capacitive sensing was obtained via a dedicated library called CapacitiveSensor.h [28], able to turn the microcontroller’s pins into a capacitive detector. In particular, the library charges the sensing plate with a very small quantity of current until the microcontroller is able to detect the signal in the plate. This process produces a quantitative measurement of the capacity of the plate in arbitrary units. A further discussion on the way this library works and its implication on the system are presented afterwards. To achieve reliable sensing, a high-value resistor (330 kΩ) was needed in pair with a metal plate made out of copper tape. On the board, there was also an accelerometer to detect the chair vibrations and a toggle button in order to register precisely the sitting activity timing. The latter feature was needed to compare the post-elaboration data and will be present only in prototypes. During each acquisition, the data of the entire experiment were firstly stored in the server and then analysed using MATLAB scripts.

### 4.1. Sensing Plate Geometry

In all the experiments described in this section, a solid wood chair with a 43 × 43 $cm^{2}$ square and 3 cm thick seat was used. A measurement session was a time frame of 13 min subdivided, respectively, in one frame of 5 min and 8 frames of one. In the first frame, the chair was left unused to simulate a setup phase; then, in altering frames a person sat and stood up, except the second-last time in which a 6 bottle of water bundle was placed on the chair. A median filter of 9 elements was implemented in the system to reduce the noise. In all these cases, the parameters observed were the mean and the square root of the variance of the signal during the difference frames, in particular during the setup phase, during the sitting phase and the mean when the water bottles were present. However, the library induces an event called auto-calibration if the measurement reaches time-out conduction: this phenomenon could be exploited in the case of false positives, such as with the water bottles, but it could be detrimental in true positive cases. The optimal parameters for our system should be a mean in the setup phase as low as possible, a reasonably high mean during the sitting frame, and as low as possible mean during the water phase, possibly with auto-calibration and variance as small as possible in all cases.

**Figure 2:**
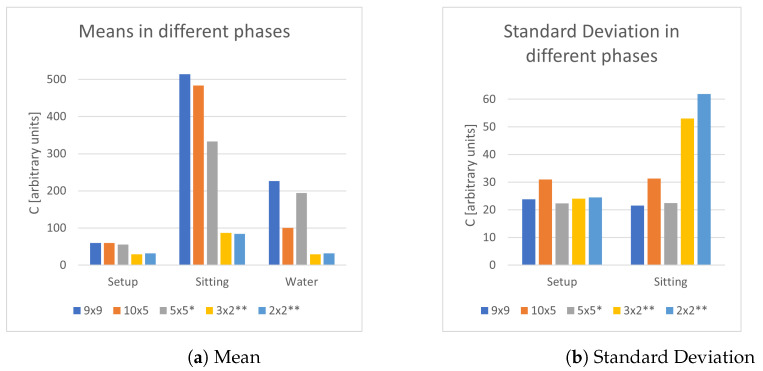
Study of different areas of sensing plates. * Auto-calibration occurred during water frames; ** Auto-calibration occurred both in water and sitting frames.

The first objective was to determine the optimal area of a single plate detector. In multiple measurement sessions, the system used a sensing plate made of copper with a decreasing area, respectively, 9 × 9, 10 × 5, 5 × 5, 3 × 2, 2 × 2 $cm^{2}$. In Figure 2, it can be noticed that the small plates have a really good mean in the setup phase, yet they fall short in the sitting phase due to auto-calibration and a very high value of the standard deviation, implying an unstable signal. Even if the higher area cases (9 × 9 and 10 × 5) have better performance across the board compared to the 5 × 5 case, they do not trigger auto-calibration in the case of water bottles, making the 5 × 5 sensing plate a reasonable trade-off in terms of performance, cost and (theoretically) energy consumption. These results can be interpreted as the linear dependence of the capacitance in terms of the area of the capacitor: smaller capacitors have bigger time constants, which could obstacle stable measurements.

**Figure 3:**
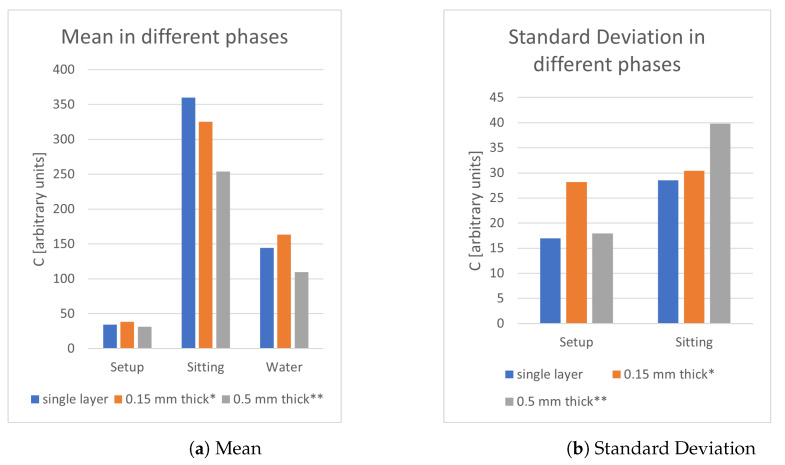
Study of different thickness of sensing plates. * Auto-calibration occurred during water frames; ** Auto-calibration occurred both in water and sitting frames.

The second objective was to determine how the thickness of the sensing plate could change the measurement process. Common aluminium foils were used with the fixed area and the thickness, respectively, of a single foil, 0.15 mm and 0.5 mm. As Figure 3 shows, the single foil case always performs better than the other two cases, even if the value does not vary significantly. This phenomenon can be attributed to the skin effect of the metals and to the bigger charge dispersion.

**Figure 4:**
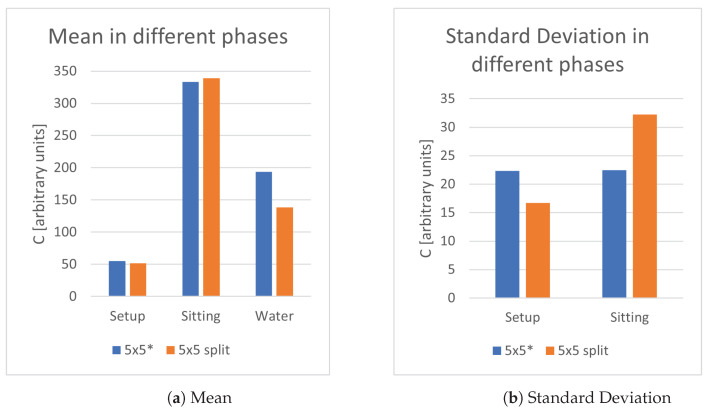
Study of split configurations. * Auto-calibration occurred during water frames.

The third objective was to determine if a similar sensor area split in smaller bits could provide better sensitivity than a unique piece, thanks to the better positioning of smaller bits in the seat. In this test, a single plate 5 × 5 $cm^{2}$ plate, 4 plates of area 2.5 × 2.5 $cm^{2}$ disposed in a square with a 9 cm side connected along the sides of the square. In both cases, the sensing plate was made of a single sheet of copper foil. According to the results in Figure 4, the split sensing plate yields better results in the case of water detection and some minor benefits in the setup and sitting phase. This result is clearly due to the better localization of the sensing plate with respect to the high mass parts of the human body.

**Figure 5:**
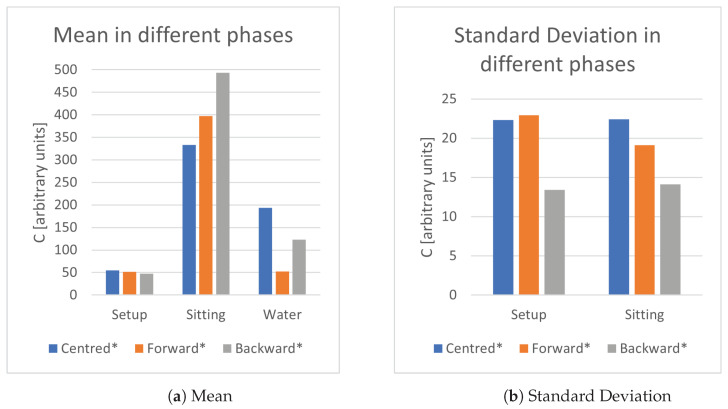
Study of different positioning of sensing plates. * Auto-calibration occurred during water frames.

The fourth objective was to determine how the positioning of the sensor under the seat affects the capacitance measurements. Three experiments were undertaken with a 4 plates sensing plate of area 2 × 2 $cm^{2}$ positioned in a square with a 9 cm side connected in the centre of the square: one with the sensor placed in the middle of the seat, one with the sensor located as forward as possible, and one with the sensor displaced as backward as possible. Figure 5 shows that there are not many differences between the forward and centred configuration when a correct sitting position is maintained. Moreover, the backward positioning results in much better signals. However, it should be noticed that in case of a not correct sitting position, e.g., when the person does not touch the backseat, the first two settings are not able to produce a clear signal, so the forward configuration is able to constitute a more robust system.

**Figure 6:**
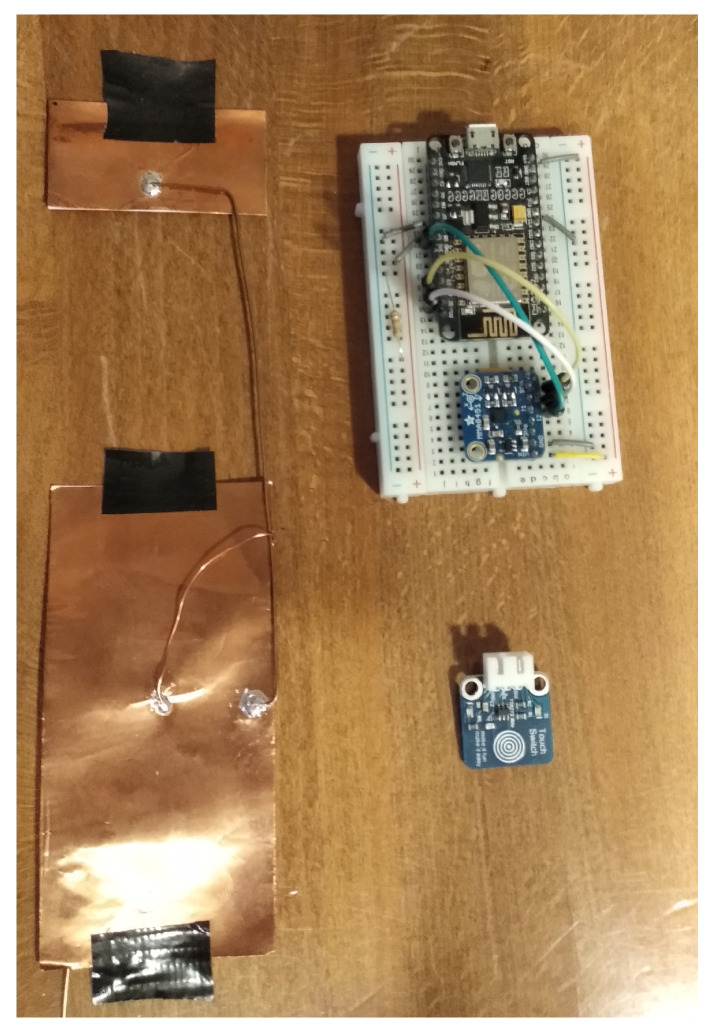
Final design of the system.

The final design also considered the possibility of the use of a cushion, a practice that is common in elderly users. Different quick tests showed how the presence of the cushion greatly reduces the intensity of the signal and increases the chances of an auto-calibration event. The final design features a bigger plate to mitigate this effect and a positioning able to sense most of the sitting positions. The proposed structure (shown in Figure 6) was composed of a 10 × 5 $cm^{2}$ plate placed vertically in the centre of the seat and another 5 × 2 $cm^{2}$ plate situated as upfront as possible, in order to have a strong signal in every possible configuration and not to risk a false negative. The total area of the design is not the main concern for battery consumption since the sampling rate is not required to assume extremely high values for prolonged periods of time.

### 4.2. Real Life Usage Performance

**Figure 7:**
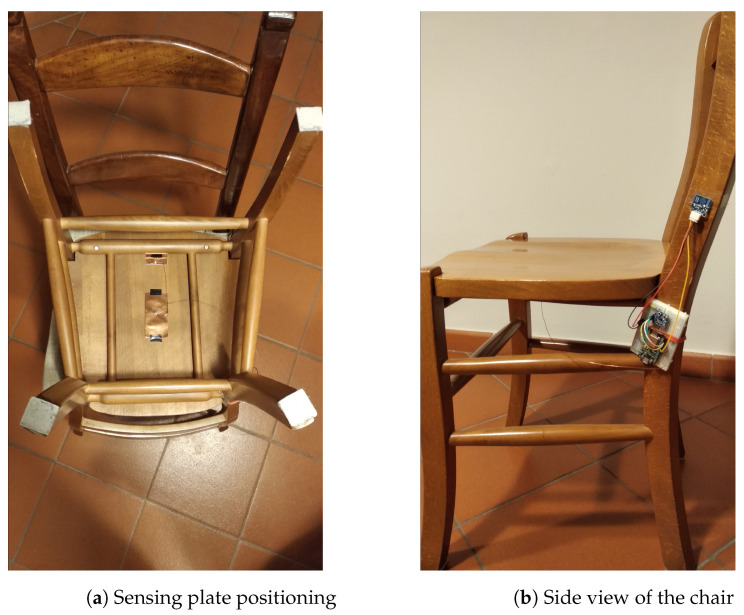
Structure of the system during the experiments.

In this section, two real environment tests of the system are presented, one during a meal and the other one in a living room scenario. The distinction between the two is necessary since it was experimentally observed that the interrupts are not always generated by the single act of sitting or standing up, especially in the latter case, but more often by the translation or rotation of the chair to reach a table. The system was equipped with the sensing plate described at the end of the last section and was mounted on a solid wood chair with a side of 43 cm and 3 cm thick seat in Figure 7. To verify the precision of the system, the data provided from the button described before were used as ground truth. The goal of the experiment was to determine if the algorithm is able to detect when a person sits on the chair during a time window of N elements, so the data obtained by the software are compared with the ground truth averaged over the N elements of the window.

**Figure 8:**
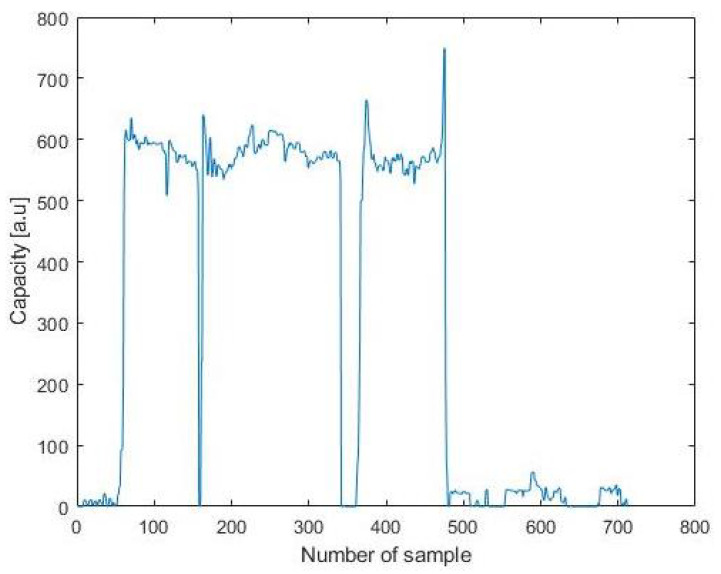
Graph of the capacity during the test.

The first experiment was performed during a dinner lasting about half an hour. The capacitive measurements are shown in Figure 8, where it can be clearly seen where the chair is used. The data were analysed and produced the confusion matrix in Table 3, where it is possible to observe the occurrences of correct (P) or incorrect (N) readings in the two possible conditions: person sitting, person not sitting. The final results show an accuracy of 97.81% and a precision of 95.2%. Most of the false negatives were due to the asynchronous act of sitting and pressing the button to record the status.

**Figure 9:**
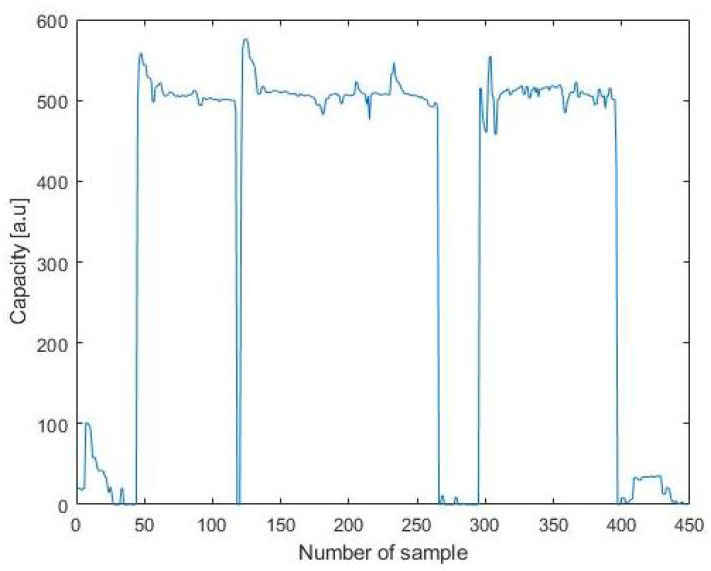
Graph of the capacity during the test.

The second experiment took place in the middle of the living room and lasted a bit more than half an hour. The capacitive measurements are shown in Figure 9, where it can be clearly seen where the chair is used. Data were analysed and produced the confusion matrix in Table 4, where it is possible to observe the occurrences of correct (P) or incorrect (N) readings in the two possible conditions: person sitting, person not sitting. Final results shows an accuracy of 98.58% and a precision of 95%, which is probably given by the fact that the interrupts were always generated when the sitting status changed. Moreover, it should be noted that similarly to the previous case, the majority of the false negatives were given by the asynchronous act of sitting and pressing the button to record the status.

Given the innovativeness of the system, it would be hard to compare it against the state of the art works on fall detection, since the system is not actually directly detecting the event of a person falling. However, it is possible to make a comparison with the results of the work of Rosato [27], since the methodology is similar to the one presented in this paper. The best results in terms of accuracy and precision were, respectively, reported as 95% and 94%. Both systems share very similar results. It is important to note that even if a pure and unbiased signal is preferable, due to the nature of the fall event, false-positives should be considered as potentially more harmful. In fact, if the system detects a person sitting while a fall has taken place, it can cause a delayed warning to the caregiver, which may lead to negative health effects. However, in the proposed system the presence of false positives is not to be considered critical. In fact, during a state transition, an interrupt signal will be generated and the sampling frequency increases dramatically. For this reason, the time window right after the transition is very short in time and any error would be quickly corrected. It is then possible to implement a looser requirement on the time response of the system leading to better results regarding false positives and false negatives.

### 4.3. Power Consumption Analysis

Further analysis has been carried out in order to estimate the power consumption of the system under general usage by dividing our client into its main parts and testing them out separately.

The workbench consisted of a power supply able to power the system at 3.3 V with stability, a small and precise resistor and a multimeter.

In order to estimate the power consumption, the multimeter measured the voltage drop across the resistor which was in series with the system and then, with the first Ohm’s law it was possible to obtain the current consumption.

At first, the consumption analysis involving the sensing element with the main MCU was carried out under different workloads.

In full sleep mode the current consumption was at around 70 μAh.

Subsequently, the MCU was programmed to continuously retrieve data from the capacitive plate, leading us to power consumption during the sensing activity of around 8 mAh with every acquisition lasting about 40 ms.

Lastly, the communication stage was tested. It must be noted that in order to obtain power consumption data, the integrated Wi-Fi card of the NodeMCU was used by continuously sending packets to a server. This process lead to a current consumption of around 100 μAh for around 10 ms per packet. Furthermore, this consideration is a rather rough approximation, indeed a Wi-Fi cart is much more energy-hungry than a BLE interface, yet, considering the considerations mentioned above, it can be argued that it is a reasonable upper limiting case estimation.

Thanks to all this, it was possible to obtain the expected power consumption over an hour during a time frame where only an interrupt is generated. Considering the current consumption during all the three stages described weighted for the amount of time they lasted obtaining around 80 μAh thanks to Equation (1). This result leads us to believe that with a rather small battery like a CR2032, the device could be active for a time frame of at least 120 days. Moreover, using a more common AA battery, the battery life could be increased up to 10 times more.

$I_{i}V$ represent the energy consumption for the given event i (sensing phase, sleep mode phase, communication phase), $\tau_{i}$ represent the amount of time the single phase lasted and $n_{i}$ account for the number of instance of phase *i* in one hour.

(1) $$E_{tot}=\frac{I_{sens}\tau_{sens}n_{sens}+I_{wifi}\tau_{wifi}n_{wifi}+I_{sleep}\tau_{sleep}}{\tau_{h}}V$$

Given all the previous experimental considerations, a question on which waiting time used in the prototype is the best one in terms of performance and consumption arises. At first, it was necessary to define a realistic use case in which the system would be tested. Empirically, a chair placed in the kitchen of the home may be used multiple times a day for brief amounts of time and twice or three times in an intensive way. The hypothesis was made that during the day, during 4 h, the chair would be used four times, during 4 h it would be used twice and for the remaining time, the chair would not be touched at all. The main requirement for the sensor is that for no more than 5 min the sensor may wrongfully detect the occupation status of the chair.

With these assumptions, it was possible to simulate on MATLAB the behaviour of the sensor in order to test different waiting functions. During the generation of the data, the possibility of measurements errors was included through the use of the confusion matrix obtained in the experiments done before.

The first case we studied was the dynamic of the original system, which can be described with the discrete linear system in (2). Different cases were done tinkering with the parameter in (2). Moreover, in the last three cases, completely new functions were used, such as a linear and a quadratic one.

(2) $$\left\{\begin{array}{c} x(0)=100\\ x(t+1)=\alpha x(t) \end{array}\right.$$

The results are shown in Table 5, in which all the considered cases result in better battery life to the detriment of the number of samples recorded. However, no configuration resulted in the wrong detection pattern for more than 5 min. Moreover, the best compromise with performance and battery life is reached with case 2, in which the linear system has its coefficient changed from 1.1 to 2. This results in a battery life gain of 15% while maintaining a decent sampling rate. Moreover, it can be argued that the linear system with α equals 2 can be implemented with a simple sum, making it computationally easier and less power-hungry.

Based on the experimental results, the proposed system proves to be a reliable solution for solid wood chairs with a flat under the seat, even with a reasonably thick cushion. These results clearly indicate that the system is usable with stools and straw woven furniture.

## 5. Conclusions

The proposed seat detection system shows great performance in terms of accuracy and speed of detection in the study case while maintaining its requirement in terms of functionality, modularity, connectivity, power management, non-intrusiveness, and non-disruptiveness.

The first step for future development would include improvements in the code, in the final engineering of the object that would contain the system, such as a printed circuit board or a modular sensing plate that could be chosen on different occasions, and in the development of the web server and related interface, possibly with some APIs that would allow the proposed system to fully interface with a Smart Home environment.

Secondly, the proposed seat detection system will be integrated to a smart home monitoring system [29] in order to provide new data to validate the proposed innovative paradigm to detect falls.

Finally, the idea of a sensor fusion approach using capacitive and accelerometer data could be applied in other elderly care cases, such as beds and sofas. In fact, with less volatile objects the system could reduce the constraints in energy and allow the use of long chains of smaller plates, maybe placed under the sheets. Such a pervasive system could theoretically provide a low invasive and effective detection for a moderate price tag.

## Figures and Tables

**Table 1:** Evaluation of different types of sensors with respect to power consumption, intrusiveness, reliability, and price.

Sensor	Sensed Variable	Power Cons.	Intrusiveness	Reliab.	Price
PIN	Vis. light	medium	high	low	high
Ultrasound	Sound	high	medium	medium	medium
PIR	Inf. light	high	medium	medium	medium
Microphones	Sound	medium	low	low	low
Load cells	Weight	medium	medium	medium	high
Accelerometer	Acceleration	low	low	medium	low
Capacitive	Capacity	medium	low	high	low

**Table 2:** Capacitive and accelerometer sensors analysis.

	MPR121	CAP1203	MMA8451	MPU6050
Normal Consumption	29 µA	500 µA	165 µA	500 µA
Power saving Cons.	3 µA	50 µA	6 µA	5 µA

**Table 3:** Confusion Matrix of the first experiment.

		Actual	Status
		**P**	**N**
Predicted	**P**	101	5
status	**N**	0	122

**Table 4:** Confusion Matrix of the second experiment.

		Actual	Status
		**P**	**N**
Predicted	**P**	38	2
status	**N**	0	101

**Table 5:** Wait function comparison.

Function	Days	Gain [%]
Linear system α = 1.1	110.7	0
Linear system α = 2	127.3	15
Linear system α = 3	129.2	16.7
Linear system *x*(0) = 1000	119.1	7.6
Quadratic	123.9	11.9
Linear	103.4	−6.5

## Data Availability

Not applicable.
